# Exosomes derived from colorectal cancer cells take part in activation of stromal fibroblasts through regulating PHLPP isoforms

**DOI:** 10.17179/excli2024-6926

**Published:** 2024-05-02

**Authors:** Fatemeh Khaloozadeh, Ehsan Razmara, Fatemeh Asgharpour-Babayian, Alireza Fallah, Reihaneh Ramezani, Fatemeh Rouhollah, Sadegh Babashah

**Affiliations:** 1 Department of Cellular and Molecular Biology, Faculty of Advanced Sciences and Technology, Tehran Medical Sciences, Islamic Azad University, Tehran, Iran; 2 Department of Medical Genetics, Faculty of Medical Sciences, Tarbiat Modares University, Tehran, Iran; 3 Research and Development Center of Biotechnology, Tarbiat Modares University, Tehran, Iran; 4 Department of Family Therapy, Women Research Center, Alzahra University, Tehran, Iran; 5 Department of Molecular Genetics, Faculty of Biological Sciences, Tarbiat Modares University, Tehran, Iran

**Keywords:** colorectal cancer, cancer associated fibroblasts, exosomes, miR-224, PHLPPs, Akt signaling

## Abstract

Given that tumor cells primarily instigate systemic changes through exosome secretion, our study delved into the role of colorectal cancer (CRC)-secreted exosomal miR-224 in stromal reprogramming and its impact on endothelial cell angiogenesis. Furthermore, we assessed the potential clinical significance of a specific signature of circulating serum-derived miRNAs, serving as a non-invasive biomarker for CRC diagnosis. Circulating serum-derived miR-103a-3p, miR-135b-5p, miR-182-5p, and miR-224-5p were significantly up-regulated, while miR-215-5p, and miR-455-5p showed a significant down-regulation in CRC patients than in healthy individuals. Our findings indicated that the expressions of CAF-specific markers (α-SMA and FAP) and CAF-derived cytokines (IL-6, and SDF-1) were induced in fibroblasts stimulated with SW480 CRC exosomes, partly due to Akt activation. As a plausible mechanism, exosomal transfer of miR-224 from SW40 CRC cells may activate stromal fibroblasts, which in turn, may promote endothelial cell sprouting. The study identified PHLPP1 and PHLPP2 as direct targets of miR-224 and demonstrated that CRC-secreted exosomal miR-224 activates Akt signaling by regulating PHLPP1/2 in activated fibroblasts, thereby affecting the stromal cell proliferation and migration. This study established a panel of six-circulating serum-derived miRNAs as a non-invasive biomarker for CRC diagnosis. Also, we proposed a supporting model in which CRC-secreted exosomal miR-224 takes part in the stromal reprogramming to CAFs partly through regulating Akt signaling. This may affect the malignant biological behavior of activated stromal cells and thereby elicit a vascular response within the microenvironment of CRC cells.

See also the graphical abstract(Fig. 1).

## Introduction

Colorectal cancer (CRC) is a prevalent malignancy that leads to a significant number of cancer-related fatalities on a global scale. Despite advancements in diagnostic and therapeutic approaches, the clinical prognosis and outcome for patients with advanced-stage CRC continue to be unsatisfactory (Buccafusca et al., 2019[9]; Zhou et al., 2022[69]). Multiple studies have uncovered that adjuvant therapies have the potential to significantly enhance the outlook of individuals with advanced CRC, eradicating residual cancer that cannot be detected (Kosmider and Lipton, 2007[30]; Zhu et al., 2023[71]). Therefore, gaining further knowledge into the molecular mechanisms that control the development and advancement of CRC is crucial for identifying new molecular targets and improving the effectiveness of therapies. 

The discovery of non-coding RNAs and the vehicles carrying them has revolutionized our understanding of cellular communication, highlighting their roles in both physiological and pathological processes. These molecules play pivotal roles in various aspects of CRC progression and metastasis (Lafitte et al., 2019[31]; Nie et al., 2021[47]). Exosomes are a class of cell-derived extracellular nanovesicles of endosomal origin that are secreted into the extracellular milieu by both tumor cells and surrounding tissue (Whiteside, 2016[61]). Exosomes derived from tumor cells have been found to carry a diverse cargo of proteins, coding and non-coding RNAs, lipids, and other bioactive molecules (Gurung et al., 2021[23]; Sun et al., 2023[52]). These molecules, upon transfer to recipient cells, can modulate various cellular processes involved in tumor progression, angiogenesis, or even immune evasion (Pakravan et al., 2017[49]; Tai et al., 2018[54]; Wang and Zhang, 2021[59]). Depending on their cargos, exosomes affect the behavior and phenotype of neighboring or distant cells, shaping the tumor microenvironment (TME), and promoting the survival and expansion of tumor cells (Ghafouri et al., 2023[19]; Pakravan et al., 2022[50]; Xu et al., 2022[65]). 

Among the various types of cargo transported by exosomes, microRNAs (miRNAs, miRs) have garnered significant attention. MiRNAs represent a distinctive group of small regulatory RNA molecules that exert control over protein translation by selectively attaching to the 3'-untranslated region (UTR) of their target mRNAs (Babashah, 2014[2]). Aberrant expression of miRNAs is commonly observed in various types of human cancers (Babashah et al., 2012[3]; Babashah and Soleimani, 2011[4]). Dysregulated miRNAs play critical roles in CRC initiation, progression, metastasis, and response to therapy (Chi and Zhou, 2016[13]; Zhang et al., 2021[68]). So as to gain comprehensive insights on the pathophysiological significance of exosomal miRNAs in CRC, certain aspects require clarification. One aspect that requires further illustration pertains to the interaction between miRNAs and certain signaling pathways in the context of tumorigenesis (Dokhanchi et al., 2021[16]; Ghahhari and Babashah, 2015[20]; Syed and Brüne, 2020[53]). Indeed, in this context, tumor-surrounding stromal cells exhibit a strong correlation with the establishment of new blood vessels named angiogenesis and possess the capacity to invade through tissues during metastasis (Lou et al., 2017[38]).

The interaction between tumor cells and TME components takes crucial part in tumorigenesis (Anderson and Simon, 2020[1]). As a major component of TME, cancer-associated fibroblasts (CAFs) play crucial roles in promoting tumor growth and progression (Ma et al., 2018[40]). Earlier studies indicated that CAFs promote tumorigenesis by inducing the angiogenesis (De Palma et al., 2017[15]; Huang et al., 2019[25]; Tang et al., 2016[56]). In this regard, higher expression of vascular endothelial growth factor (VEGF) as the predominant angiogenic factor was found as a poor prognostic marker for survival in CRC patients (Cao et al., 2009[11]). Hyperactivation of Akt signaling plays a crucial role during multistage processes of CRC tumorigenesis and angiogenesis (Jiang and Liu, 2009[26]). Pleckstrin homology (PH) domain leucine-rich repeat protein phosphatases (PHLPP) belong to a family of Ser/Thr protein phosphatases that consists of PHLPP1 and PHLPP2 isoforms. The PHLPP 1/2 phosphatases possess the ability to directly dephosphorylate Akt, leading to the inhibition of Akt signaling activity and facilitating tumor growth (Brognard and Newton, 2008[7]; Grzechnik and Newton, 2016[22]; Li et al., 2011[35]). Loss of both PHLPP isoforms occurs at high frequency in CRC patients (Liu et al., 2009[36]). The primary objective of the present study was to establish a panel of six-circulating miRNA signature in serum as a non-invasive biomarker for CRC diagnosis. We also aimed to investigate how tumor cells induce stromal reprogramming to CAF, thereby affecting tumor angiogenesis. Our findings unveiled a possible mechanism through which CRC-secreted exosomal miR-224-5p selectively activate Akt signaling by regulating PHLPP isoforms.

## Materials and Methods

### Clinical samples

A total of 40 CRC patients and 30 healthy individuals were included in this study. The study received approval from the ethics committee of Islamic Azad University, Tehran, Iran, and each participant signed the informed consent. None of the patients had a history of endocrine, immune, or metabolic disorders. The disease diagnosis was confirmed by histopathological analysis of surgically resected tumor samples. A record of the clinicopathological features of CRC patients is summarized in Supplementary information, Table 1. To isolate serum, each participant's peripheral blood samples were subjected to centrifugation at 1000×g for 10 min at 4 °C, and the serum samples were then stored at 80 °C.

### Cell culture

The human colon cancer cell lines (SW480 and Caco2) and umbilical vein endothelial cells (HUVECs) and bone marrow stromal-derived fibroblasts (HS5) were obtained from Pasteur Institute of Iran (Tehran, Iran). All cell lines were maintained in Dulbecco's modified Eagles medium (DMEM) supplemented with 10 % fetal bovine serum (FBS) and 1 % antibiotics (100 U/ml penicillin, and 100 μg/ml streptomycin; all from Gibco, USA) at 37 °C in a humidified incubator containing 5 % CO_2_.

### Isolation and characterization of exosomes 

The CRC cells were cultured in the medium complemented with 10 % exosome-depleted serum (EXO-FBS; System Biosciences) for 48 h. After reaching 80-90 % confluency, the culture supernatants were harvested and subjected to differential centrifugation. The residual cells and cellular debris were eliminated by subjecting the pooled culture supernatant to centrifugation at 300×g for 10 min and then at 10,000×g for 30 min, respectively. Finally, exosomes were pelleted using the ExoQuick Exosome Precipitation Solution kit (System Biosciences) according to manufacturer's instructions and resuspended in PBS. The exosome concentrations were determined indirectly by measuring the amounts of exosome-associated proteins using a Bradford assay. Exosome-specific surface markers CD9 and CD81 were detected by western blotting as previously described (Masoumi-Dehghi et al., 2020[45]). The size and morphology of isolated exosomes were observed using transmission electron microscopy (TEM, LEO 906 Zeiss 100 kV, Germany) and scanning electron microscopy (SEM, KYKYEM3200, China). 

### Cellular uptake of purified exosomes

To monitor the internalization of exosomes into fibroblasts, a PKH26 red fluorescent labeling kit (Sigma, St. Louis, MO, USA) was used to label purified exosomes. Briefly, 4 μl of PKH26 dye were mixed with around 25 μg exosomes in 1 mL of diluent C and incubated at room temperature for 20 min. A negative control was used to detect PKH26 dye carryover. BSA was added and the mixture with PBS to stop the labeling process. The labelled exosomes were purified as described above. The fluorescence uptake of the labeled exosomes was monitored using a spectral confocal microscope (Nikon Eclipse TiE) after they were incubated with HUVECs at 37 °C for 12 h. For nuclear staining, DAPI (4′, 6-diamidino-2-phenylindole, Sigma-Aldrich) was used before taking any images.

### Functional enrichment analyses

In order to investigate the regulatory functions of miRNAs and identify significant Kyoto Encyclopedia of Genes and Genomes (KEGG) pathways, we employed the DIANA-miRPath v3.0 online tool (accessible at https://dianalab.e-ce.uth.gr/html/mirpathv3/). We also used miRTarBase v7.0 to identify the verified target pathways of candidate miRNAs. The miRTargetLink Human algorithm (https://ccb-web.cs.uni-saarland.de/mirtargetlink/) was also employed to ascertain robustly supported candidate miRNAs based on experimental evidence and generate an interaction network. In order to predict the potential targets of miR-224, we used TargetScan (http://www.targetscan.org/). 

### Transient transfection assays 

The experiments involved transfecting cells with either a miR-224-5p mimic or a negative control at a concentration of 25 nM, or an inhibitor of miR-224-5p or a scramble at a concentration of 50 nM using Lipofectamine RNAiMAX. In addition, for dual luciferase reporter assay, the cells were co-transfected with psi-CHECK2 luciferase reporter vectors containing the 3′-UTR of PHLPP1or PHLPP2 and either the miR-224-5p mimic or the negative control. After 4 hours, the cell lysate was collected and added to a 96-well plate, and the luciferase activity was measured using the Dual-Luciferase Reporter Assay System (Promega Corp., USA).

### Western blotting

To extract total protein from cells or exosomes, a lysis buffer (50 mM Tris-HCl at pH 8.0, 150 mM NaCl, 2 mM EDTA, and 0.1 % NP-40) containing a protease inhibitor cocktail was used. The proteins were separated by 10 % SDS-polyacrylamide gel electrophoresis (PAGE) and transferred to polyvinylidene difluoride (PVDF) membranes. To block the membranes, 5 % skim milk was used at room temperature for 1 h. Primary antibody incubation was carried out at 4 °C for 12 h, followed by horseradish peroxidase (HRP)-conjugated secondary antibody incubation at room temperature for 1 h. The membranes were subjected to chemiluminescence using an ECL Kit (Amersham, UK). β-actin was used as a loading control.

### Enzyme-linked immunosorbent assay 

Quantitative measurements of secreted VEGF were performed on the culture supernatants of exosome-treated fibroblasts, after 48 h, using enzyme-linked immunosorbent assay (ELISA, ab100662, Abcam) according to the manufacturer's instructions. All cell culture supernatants were used undiluted.

### RNA extraction and real-time quantitative PCR 

Total RNA was extracted from cells, exosomes, or serum using TRIzol reagent (Invitrogen) according to the manufacturer's recommendation and treated with RNase-free DNase (Fermentase, Lithuania). RNA was then reverse-transcribed into complementary DNA (cDNA) using the PrimeScript 1st strand cDNA synthesis kit (TAKARA, Japan). To quantify miRNAs, the poly-(A)-tailed RNAs were reverse-transcribed using an anchored oligo (dT) primer as described previously (Bitaraf et al., 2020[6]). Quantitative RT-PCR (qRT-PCR) was conducted on an ABI Step One Sequence Detection System (Applied Biosystems, Foster City, CA, USA) using SYBR Premix Taq™ II (TAKARA, Japan). Fold changes in mRNA or miRNA expression levels were calculated by normalizing to glyceraldehyde-3-phosphate dehydrogenase (GAPDH) or RNU6B (U6), respectively, using the comparative threshold cycle method (Livak and Schmittgen, 2001[37]).

### Proliferation assay

Fibroblasts were transfected with miR-224 inhibitor or exposed to varying concentrations of exosomes. HUVECs were stimulated with the conditioned media of fibroblasts incubated with CRC-cell derived exosomes alone or pre-transfected with miR-224 inhibitor. Viable cells were counted in triplicate using a trypan blue exclusion assay at different time points following incubation or transfection. The cell counts were compared to those of the control cells.

### Transwell migration assay

Fibroblasts incubated with 100 µg/mL exosomes or transfected with miR-224-3p inhibitor were starved in FBS-free medium. The cells were seeded at a density of 1×10^5^ cells/mL into the upper transwell chambers with a pore size of 8 µm (Corning, USA). The bottom part of the chambers was filled with medium containing 10 % FBS, serving as chemoattractant. Following 48 h, the cells migrated through the membrane and stuck to the lower surface of the membrane was stained using 1 % crystal violet, imaged and counted. The obtained results were averaged based on three randomly selected visual fields.

### Fibrin gel bead assay 

To evaluate endothelial sprouting *in vitro*, a three-dimensional fibrin gel bead assay was done as previously described (Welch-Reardon et al., 2014[60]). Briefly, HUVECs were stimulated with the conditioned media of fibroblasts incubated with CRC-cell derived exosomes alone or pre-transfected with miR-224 inhibitor. The cells were allowed to attach to Cytodex-3 microcarrier beads and embedded in a fibrin matrix in 24-well plates at about 150 beads per well. At least five beads were analyzed for each condition in each experiment.

### Statistical analysis

Results were expressed as the mean ± standard deviation (SD) of at least three experiments. To determine statistical significance, Student's t-test was used to compare two groups, while one-way or two-way analysis of variance (ANOVA) was used to compare three or more groups. A p-value of less than 0.05 was considered statistically significant.

## Results

### Expression analysis of CRC-secreted miRNAs and their clinical significance 

**Figure 1:**
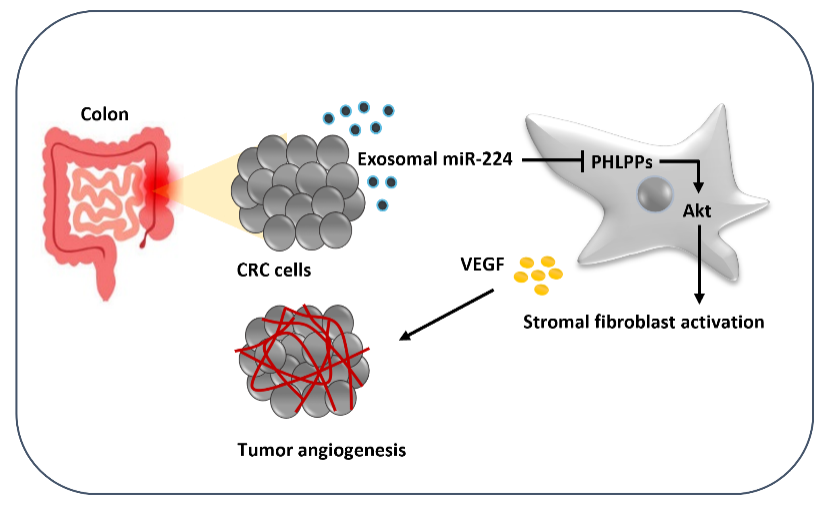
Graphical abstract

**Figure 2:**
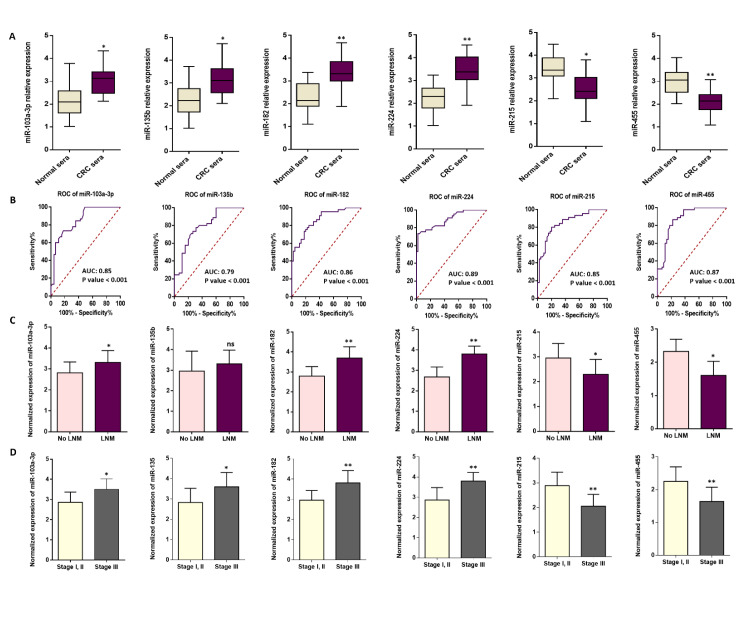
The expression levels of six serum-derived circulating miRNAs in CRC patients and healthy controls. A. The expression levels of hsa-miR-103a-3p (*p-value < 0.05), has-miR-135b-5p (*p-value < 0.05), hsa-miR-182-5p (**p-value < 0.01), and hsa-miR-224-5p (**p-value < 0.01) were higher in CRC patients than in healthy individuals; on the other hand, the levels of serum hsa-miR-215-5p (*p-value < 0.05) and hsa-miR-455-5p (**p-value < 0.01) were down-regulated in CRC serum samples than in healthy individuals. B. Receiver operating characteristics (ROC) curves analysis with 95% Confidence Interval for sensitivity/specificity to evaluate the diagnostic accuracy of each circulating miRNA. Accordingly, miR-103a-3p (Area under curve, AUC: 0.85), miR-135-5p (AUC: 0.79), miR-182-5p (AUC: 0.86), and miR-224-5p (AUC: 0.89), miR-215-5p (AUC: 0.85), and miR-455-5p (AUC: 0.87) exhibited high diagnostic performance in distinguishing individuals with CRC from healthy controls (*p*-value < 0.001). C. The expression levels of serum-derived circulating miRNAs in CRC patients with positive and negative lymph node metastasis (LNM). While the higher expression levels of serum miR-103a-3p (*p-value < 0.05), miR-182-5p (**p-value < 0.01), and miR-224-5p (**p-value < 0.01) were associated with LNM status, the expression levels of serum miR-215-5p (*p-value < 0.05) and miR-455-5p (*p-value < 0.05) were lower in LNM positive CRC samples compared to serum samples derived from patients with No LNM. Besides, hsa-miR-135b did not show any correlation with LNM status (*p *value: not significant, ns). D. The expression levels of serum-derived circulating miRNAs in association with TNM stages in CRC patients. The expression levels of serum miR-103a-3p (*p-value < 0.05), miR-135b-5p (*p-value < 0.05), miR-182-5p (**p-value < 0.01), and miR-224-5p (**p-value < 0.01) were higher in advanced stage of CRC tumorigenesis. On the other side, the levels of serum miR-215-5p (**p-value < 0.01) and miR-455-5p (**p-value < 0.01) were up-regulated in early stages of CRC development.

According to the previous findings that aimed to find non-invasive markers for CRC management, we nominated different miRNAs. The candidate miRNAs (i.e., hsa-miR-103a-3p, hsa-miR-135b-5p, hsa-miR-182-5p, hsa-miR-224-5p, hsa-miR-215-5p, and hsa-miR-455-5p) were selected from kinds of literature by considering their possible roles in CRC pathogenesis. The functional enrichment analysis implied that the candidate miRNAs play important roles during cancer development and physiological conditions (Supplementary information, Figure 1). To explore the diagnostic significance of circulating miRNAs, the expression levels of each candidate miRNA were evaluated in serum samples obtained from both CRC patients and healthy individuals. The results obtained from RT-qPCR demonstrated a significant up-regulation of miR-103a-3p, miR-135b-5p, miR-182-5p, and miR-224-5p in serum samples collected from patients with colorectal cancer (CRC) compared to those from healthy individuals. On the other hand, the expression of miR-215-5p and miR-455-5p were found to be down-regulated in CRC than normal samples (Figure 2A(Fig. 2)). To better assess the diagnostic accuracy of circulating miRNAs, receiver operating characteristic (ROC) curves were generated. Accordingly, the area under the curves (AUC) indicated high diagnostic performance of each circulating miRNA in distinguishing individuals with CRC from healthy controls (*p*-value < 0.001; Figure 2B(Fig. 2)). Of clinical relevance, a correlation comparison between the expression of miRNAs and the incidence of lymph node metastasis (LNM) in patients diagnosed with CRC was conducted. LNM-positive patients exhibited elevated levels of miR-103a-3p, miR-182-5p, and miR-224-5p; however, a statistically significant decrease was observed in the expression of miR-215-5p and miR-455-5p in relation to LNM status. Nonetheless, the expression of miR-135b did not show any statistically significant differences between positive and negative LNM patients (Figure 2C(Fig. 2)). As a precautionary measure, we also examined the potential correlation between the expression of candidate miRNAs and TNM values. Our findings revealed an elevation in the expression levels of miR-103a-3p, miR-135b-5p, miR-182-5p, and miR-224-5p in advanced stages, i.e., TNM stage III. Conversely, patients in the late stages significantly exhibited a decrease in the expression of miR-215-5p and miR-455-5p (Figure 2D(Fig. 2)). Essentially, these data provide evidence of an association between the expression of our candidate circulating miRNAs and LNM and TNM clinicopathological features in CRC. 

### Exosomes released by CRC cells contribute to activation of stromal fibroblasts partly through Akt signaling

**Figure 3:**
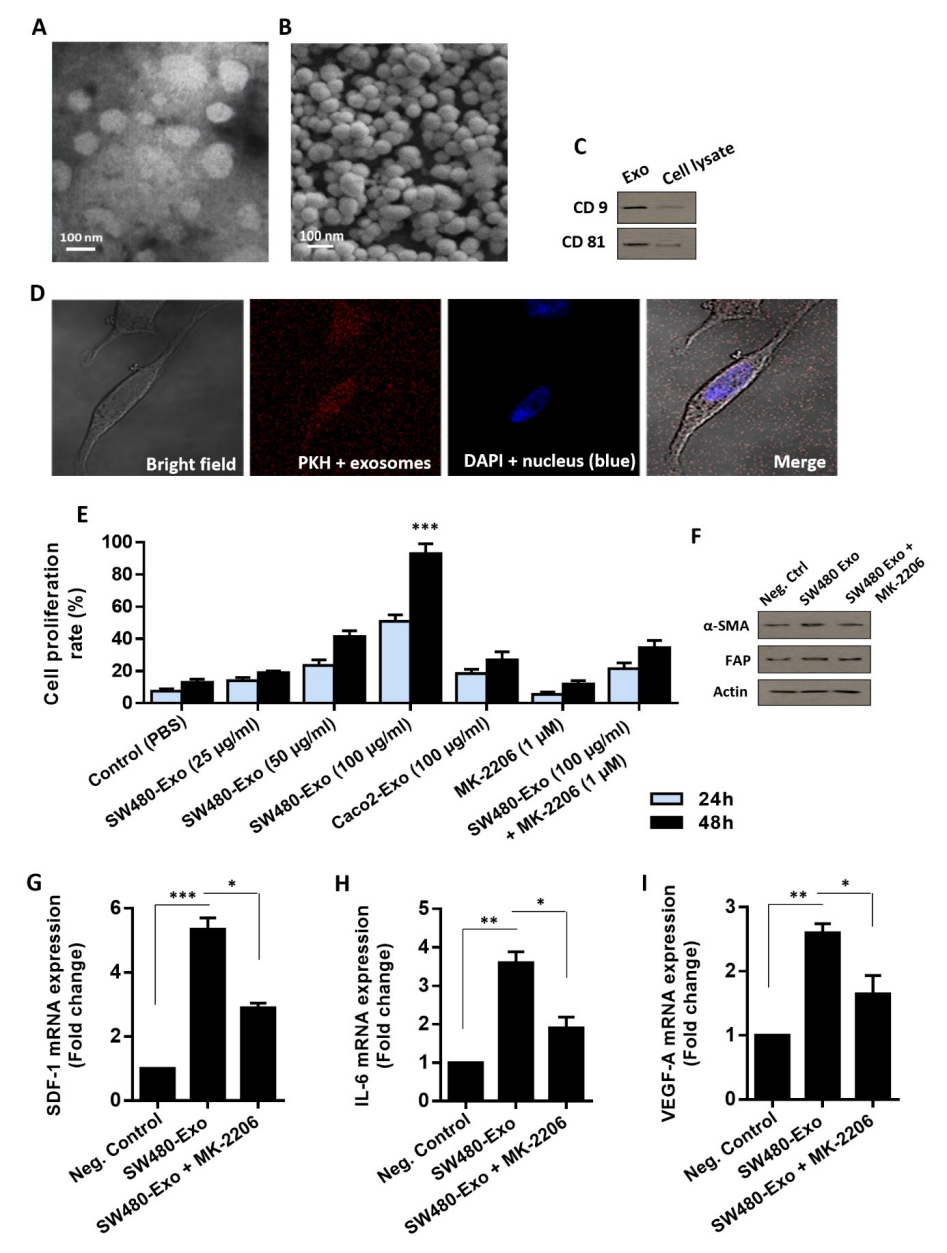
Exosomes derived from SW480 CRC cells take part in the activation of stromal fibroblasts in part through Akt signaling. Transmission (A) and scanning (B) electron micrographs of purified exosomes derived from the culture media of CRC cells depicting spherical and membrane-encapsulated particles with diameters ranging from 50 to 150 nm. C. Detection of the exosome-specific markers CD9 and CD81 in purified exosomes obtained from CRC cells as demonstrated by Western blotting. D. Internalization of PKH-labeled CRC exosomes into fibroblast. A red fluorescence in the cytoplasm of fibroblast indicates that CRC cell exosomes are readily taken up by fibroblasts. The nuclear staining was performed by using DAPI. E. Different concentrations of SW480-Exo (25, 50, and 100 μg/mL) had promoting effects on the proliferation rate of stromal fibroblasts in a dose- and time-dependent manner. Although exosomes (100 µg/mL) derived from SW480 CRC cells exhibited the most promoting effect of the stromal fibroblast proliferation; however, administration of the AKT inhibitor MK-2206 (1 μM) considerably inhibited the proliferative effects of CRC-Exo on stromal cells. F. Western blot analysis indicated a significant induction in protein levels of CAF-specific markers α-SMA and FAP in stromal cells, 48 h after treatment with 100 µg/mL SW480 exosomes. Results suggested that MK-2206 treatment (1 μM) partly diminished the promoting effects of SW480-Exo on the protein levels of CAF-specific markers in stromal cells. Actin was used as an endogenous loading control. Western blot images are representative of at least three independent experiments. G-I. The mean normalized ratios for SDF-1 (G), IL-6 (H), and VEGF-A (I) transcript levels were measured by RT-qPCR, 48 h after incubation. Results revealed that the transcript levels were considerably increased in stromal cells incubated with 100 μg/mL SW480 exosomes, compared to negative control. Importantly, inhibiting AKT activity through MK-2206 treatment (1 μM) partially prevented the promoting effects of SW480 exosomes on SDF-1, IL-6, and VEGF-A transcript levels, proposing that the effect of CRC exosomes on the activation of stromal fibroblasts partly depends on Akt activation. Columns, mean of three different experiments; bars, SD. *P-value < 0.05, **P-value < 0.01, ***P-value < 0.001.

In accordance with the guidelines provided by the International Society for Extracellular Vesicles (ISEV), the isolated EVs originating from the conditioned media of SW480 cells displayed a spherical-shaped structure, falling within the diameter range of 50-150 nm. This morphology assessment was confirmed through electron microscopy analysis (Figure 3A, B(Fig. 3)). Consistently, the western blotting assay demonstrated the presence of established exosome-specific markers (i.e. CD9 and CD81) within the isolated EVs (Figure 3C(Fig. 3)). After careful examination of the morphology, size, and protein markers, it became evident that the vesicles isolated from the conditioned media of SW480 cells corresponded to the characteristics of “Exosomes.” To investigate the role of CRC-derived exosomes on the tumor stroma, we examined the uptake of CRC-derived exosomes by the bone marrow stromal-derived fibroblasts. To put this on the experiment, we prepared PKH26-labeled exosomes that were isolated from SW480 CRC cells and, subsequently, fibroblasts were incubated with 100 μg/mL of these labeled exosomes for 12 h. Through confocal microscopy analysis, red fluorescence was observed in the cytoplasm and perinuclear region of fibroblasts, confirming the successful internalization of CRC-derived exosomes (CRC-Exo) into the cytoplasm of fibroblasts (Figure 3D(Fig. 3)). 

It is well-recognized that tumor cells cause stromal fibroblasts to become activated and produce a number of autocrine and/or paracrine cytokines and growth factors that facilitate tumors grow (Wu et al., 2021[62]). As activated stromal fibroblasts exhibit a relatively higher proliferation rate, we first examined whether CRC cell-derived exosomes had an impact on the proliferation of stromal fibroblasts. As shown in Figure 3E(Fig. 3), our findings revealed that SW480 CRC cell-derived exosomes significantly promoted cell growth in a time- and dose-dependent manner.

However, no such promoting effect of exosomes derived from non-metastatic CRC cell line Caco2 on stromal cell proliferation was observed. Given activation of Akt signaling is involved in the stromal reprogramming to CAFs in TME (Fang et al., 2023[17]), we sought to investigate whether Akt signaling is involved in CRC-Exo-mediated stromal activation. To put this on experiment, the Akt activity in stromal fibroblasts was inhibited by MK-2206, which is clinically used for the treatment of metastatic CRC (ClinicalTrials.gov identifier: NCT01802320). Our observations indicated that although SW480 CRC-Exo (100 µg/mL) enhanced the proliferation rate of fibroblasts, MK-2206 treatment (1 μM) inhibited the promoting effects of SW480 CRC-Exo on stromal cell proliferation (Figure 3E(Fig. 3)). Moreover, having shown that CRC-secreted exosomes interact with fibroblasts, we sought to investigate whether theses exosomes play a part in inducing the stromal reprogramming to CAFs. As shown in Figure 3F(Fig. 3), western blot analysis revealed that the significant induction in protein levels of CAF-specific markers α-smooth muscle actin (α-SMA) and fibroblast-activation protein (FAP) in fibroblasts induced by 100 µg/mL SW480 CRC-Exo partly depends on Akt activation. Furthermore, we analyzed the expression of CAF-derived cytokines (Interleukin-6, IL-6, and stromal-derived factor-1, SDF-1) and found that their transcript levels were increased in the SW480-Exo-tread stromal cells (Figure 3G, H(Fig. 3)). The transcript level of VEGF was also found to be increased in SW480-Exo-induced stromal fibroblasts (Figure 3I(Fig. 3)). Notably, we found that while exosomes released by metastatic CRC cells led to an up-regulation of markers specific to activated stromal fibroblasts and enhanced expression of CAF-derived cytokines and VEGF, the administration of MK-2206 (1 μM) impeded these effects to some extent (Figure 3 G-I(Fig. 3)). Altogether, these findings proposed that exosomes released by CRC cells interact with the stromal-derived fibroblasts and contribute to their activation partly through Akt signaling.

### Exosomal transfer of miR-224 from SW40 CRC cells mediates activation of stromal fibroblasts

**Figure 4:**
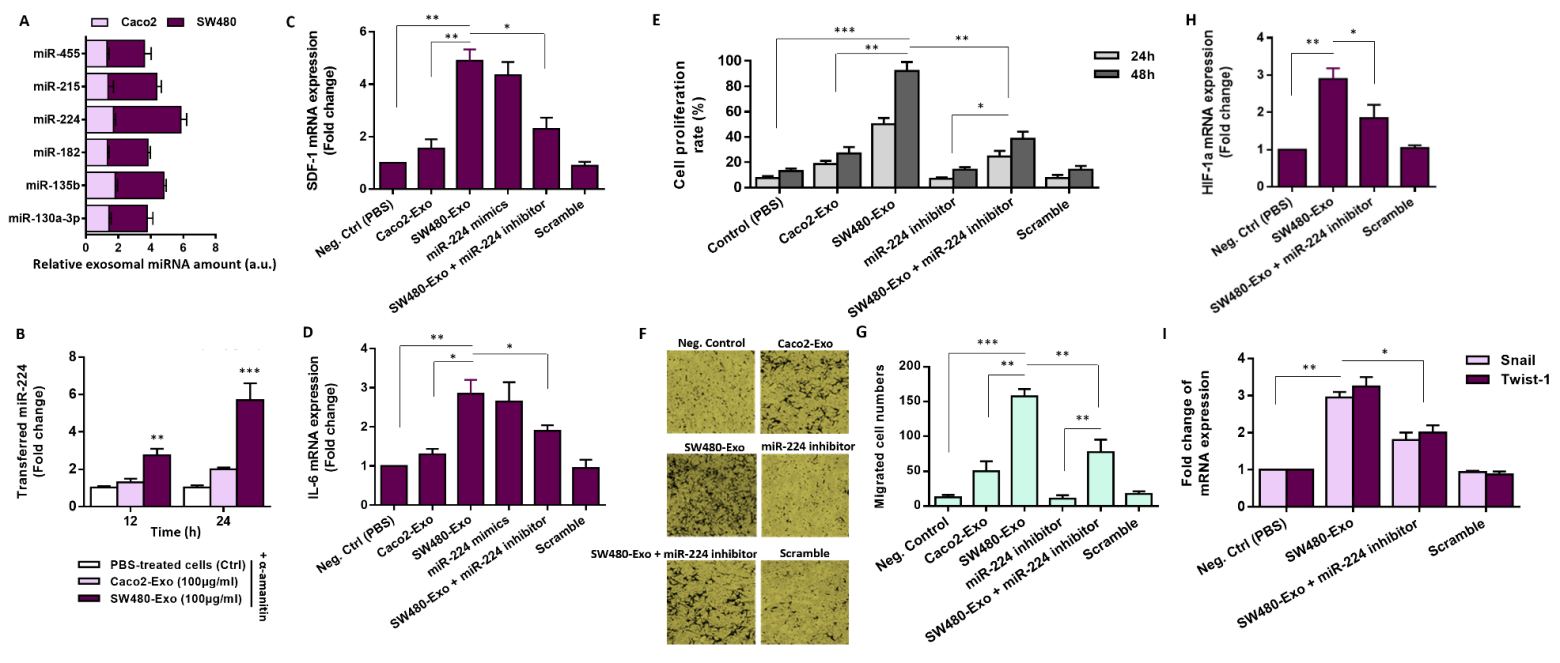
Exosome derived from aggressive CRC cells affects the malignant biological behavior of activated fibroblasts partly through transferring miR-224. A. The expression levels of candidate miRNAs were examined in exosomes derived from two different types of colorectal cancer cell models: SW480, which is metastatic, and Caco2, which is non-metastatic. To determine the relative changes in expression, fold changes were calculated by comparing the normalized mean differences between the exosomes and their respective donor cells. RT-qPCR results revealed that miR-224 was highly expressed by SW480 cells but not in the same amount in Caco2 cells. B. Intercellular transfer of miR-224-5p by CRC exosomes was evaluated by RT-qPCR in stromal cells at 12 and 24 h time points after incubation. Fibroblasts were pre-treated with RNA polymerase inhibitor α-amanitin for 10 h before incubation with CRC exosomes. The cells incubated with α-amanitin and PBS were used as negative control. RT-qPCR results demonstrated that miR-224 is transferable from SW48 CRC cells into fibroblasts through exosomes in a time-dependent manner. C, D. The mean normalized ratios for SDF-1 (C) and IL-6 (D) transcript levels were measured by RT-qPCR, 48 h after incubation. Results revealed that the transcript levels were considerably increased in fibroblasts incubated with 100 μg/mL SW480 exosomes, compared to negative control. Also, transfection of miR-224 mimic into stromal cells recapitulated the promoting effects of SW480-Exo on the transcript levels of CAF cytokines but miR-221 inhibitor partially abrogated the promoting effects of SW480 exosomes on CAF cytokines in fibroblasts. E. Exosomes (100 µg/ml) derived from SW480 CRC cells considerably augment the proliferation rate of fibroblasts in a time-dependent manner. However, incubation of fibroblasts with 100 µg/ml exosomes derived from non-metastatic Caco2 CRC cells did not significantly affect the proliferation rate of fibroblasts compared to the control cell group. Importantly, transfecting fibroblast with miR-224 inhibitor significantly antagonized the proliferative effects of SW480 exosomes. F, G. Enhanced migration of stromal fibroblasts incubated with 100 μg/mL of SW480 exosomes, compared with the corresponding control cells. F. Representative photomicrographs of the migration potential of fibroblasts in different conditions after 24 h of incubation, assessed using the transwell assay. G. Quantitative assessment of migrated cells showed that fibroblasts incubated with 100 μg/mL SW480 exosomes exhibited a significantly higher migration potential compared with negative controls or those of cells incubated with exosomes derived from non-metastatic cell line Caco2. Results identified miR-224 as a crucial factor in mediating promoting effects of SW480 exosomes on stromal cell migration because reducing its levels diminished the migratory impact of SW480 exosomes on fibroblasts. H. The mean normalized ratios for HIF-1α transcript level were measured by RT-qPCR, 48 h after incubation. Results showed that although SW480 CRC-Exo (100 μg/mL) exhibited an enhancing effect on the transcript level of HIF-1α, this effect was partially reduced when fibroblasts were transfected with miR-224 inhibitor. I. The mean normalized ratios measured for EMT markers Snail and Twist-1 indicated that the enhancing effects of SW480 exosomes were partially abolished when fibroblasts were transfected with miR-224 inhibitor. Columns, mean of three different experiments; bars, SD. *P-value < 0.05, **P-value < 0.01, ***P-value < 0.001.

It is now well-recognized that tumor-derived exosomes tend to be enriched with miRNAs (Cheng et al., 2014[12]). Hence, we first quantified the levels of the candidate miRNAs in exosomes derived from SW480 cells, which represent a metastatic cell model, and Caco2 cells, as a non-metastatic CRC cell model. The fold changes in miRNA expression levels determined by comparing the normalized mean differences between exosomes and their corresponding donor cells indicated a higher level of these miRNAs, particularly miR-224, in the metastatic SW480 cells when compared to Caco2 cells (Figure 4A(Fig. 4)). Since exosomes facilitate the communication between tumor cells and their surrounding stromal cells by transferring functional small RNAs (Tan et al., 2020[55]), we hypothesized that CRC-secreted exosomal miR-224 may mediate the activation of fibroblasts. To examine the exosomal transfer of our candidate miRNA, we first measured the level of miR-224-5p in fibroblasts at different time intervals after being treated with 100 µg/mL CRC-Exo and compared to control cells. As shown in Figure 4B(Fig. 4), a gradual increase in the level of miR-224 was observed in fibroblasts following incubation with SW480 CRC-Exo, while the level of this miRNA did not show a significant difference in the control groups. Notably, pre-treating the recipient cells with α-amanitin, an inhibitor of RNA transcription, did not hinder the increased level of miR-224-3p in fibroblasts after incubating to SW480 CRC-Exo. This suggests that the increased levels of miR-224-3p in fibroblasts did not originate from the induced transcription of endogenous miR-224-5p and, in essence, miR-224-5p originates from exosomes released by SW480 CRC cells.

We next analyzed whether CRC-secreted exosomal miR-224 takes part in activation of stromal fibroblasts. Importantly, RT-qPCR results indicated that treatment of fibroblasts with 100 µg/mL SW480 exosomes augmented the mRNA levels of IL-6, and SDF-1 than those of cells incubated with Caco-Exo or control vehicle PBS. Similarly, when fibroblasts were transfected with miR-224 mimic (Supplementary information, Figure 2A), a significant increase in the expression levels of IL-6, and SDF-1 was observed than the cells transfected with scramble negative control (Figure 4C, D(Fig. 4)). To further validate the role of exosomal miR-224 in fibroblast activation, we utilized miR-224 inhibitor that inhibits the expression of miR-224 (Supplementary information, Figure 2B). As expected, our results revealed that the enhancing effects of SW480 exosomes on the transcript levels of CAF-derived cytokines were abolished when fibroblasts were transfected with miR-224 inhibitor (Figure 4C, D(Fig. 4)). Overall, as miR-224 overexpression recapitulated the promoting effects of SW480-Exo on the transcript levels of CAF-derived cytokines, it is proposed that exosomal transfer of miR-224 contributes to activation of stromal fibroblasts. 

### CRC-secreted exosomal miR-224 affects the malignant biological behavior of activated fibroblasts

Activated fibroblasts can undergo EMT, the process in which fibroblasts lose their characteristics and acquire mesenchymal cell properties, such as increased proliferative and migratory abilities (Lamouille et al., 2014[32]). Akt signaling can affect the EMT (Xu et al., 2015[64]). As our findings point to targeting PHLPP 1/2 links miR-224 to the oncogenic Akt signaling, we sought to investigate whether exosomes containing miR-224 released from CRC cells can affect the malignant phenotype of activated stromal fibroblasts. To investigate the impact of CRC-secreted exosomal miR-224 on stromal cell proliferation and migration, fibroblasts were incubated with 100 µg/mL exosomes derived from metastatic SW480 cells or non-metastatic Caco2 cells. Our findings revealed that fibroblasts incubated with SW480 CRC-Exo, but not Caco2-Exo, exhibited an enhanced proliferation at different time intervals (Figure 4E(Fig. 4)). Besides, inhibiting miR-224 significantly decreased the proliferation of stromal cells. Notably, inhibiting miR-224 impeded the proliferative effects of SW480-Exo to some extent in fibroblasts (Figure 4E(Fig. 4)). As shown in Figure 4F(Fig. 4), incubating stromal cells with 100 μg/mL SW480 exosomes led to an enhancement in the migratory capacity of the treated fibroblasts; however, no such enhancing effect of Caco2 exosomes in the same concentration on fibroblast migration was observed. Also, the transwell migration assay indicated a significant reduction in fibroblast migration after inhibiting miR-224. Notably, the SW480 exosome treatment could not considerably restore the migration capabilities of stromal cells transfected with miR-224 inhibitor (Figure 4F, G(Fig. 4)). Overall, these findings indicate that exosomes derived from aggressive CRC cells may promote stromal cell proliferation and migration by transferring of miR-224. Given that the role of hypoxia-inducible factor (HIF)-1α in EMT is mainly achieved through transcriptional regulation (Fang et al., 2023[17]), we examined the effects of SW480 CRC cell-derived exosomes on the expression levels of HIF-1α and EMT markers (Snail and Twist). Results indicated that treating fibroblasts with 100 μg/mL SW480 exosomes caused a significant increase in the transcript levels of these genes (Figure 4H, I(Fig. 4)). To rule out the possibility that the observed effect was attributed to the functions of exosomal miRNAs other than miR-224, we transfected fibroblasts with miR-224 inhibitor. RT-qPCR results indicated that pre-transfection of fibroblasts with miR-224 inhibitor diminished the promoting effects of SW480 CRC-Exo on the transcript levels of HIF-1α and EMT markers after 48 h (Figure 4H, I(Fig. 4)). Altogether, it seems that CRC-secreted exosomal miR-224 can specifically lead to an increase in the expression of EMT-related markers, thereby affecting the malignant biological behavior of activated fibroblasts.

### miR-224, shuttled by CRC exosomes, regulates Akt signaling by targeting PHLPP isoforms in activated fibroblasts

**Figure 5:**
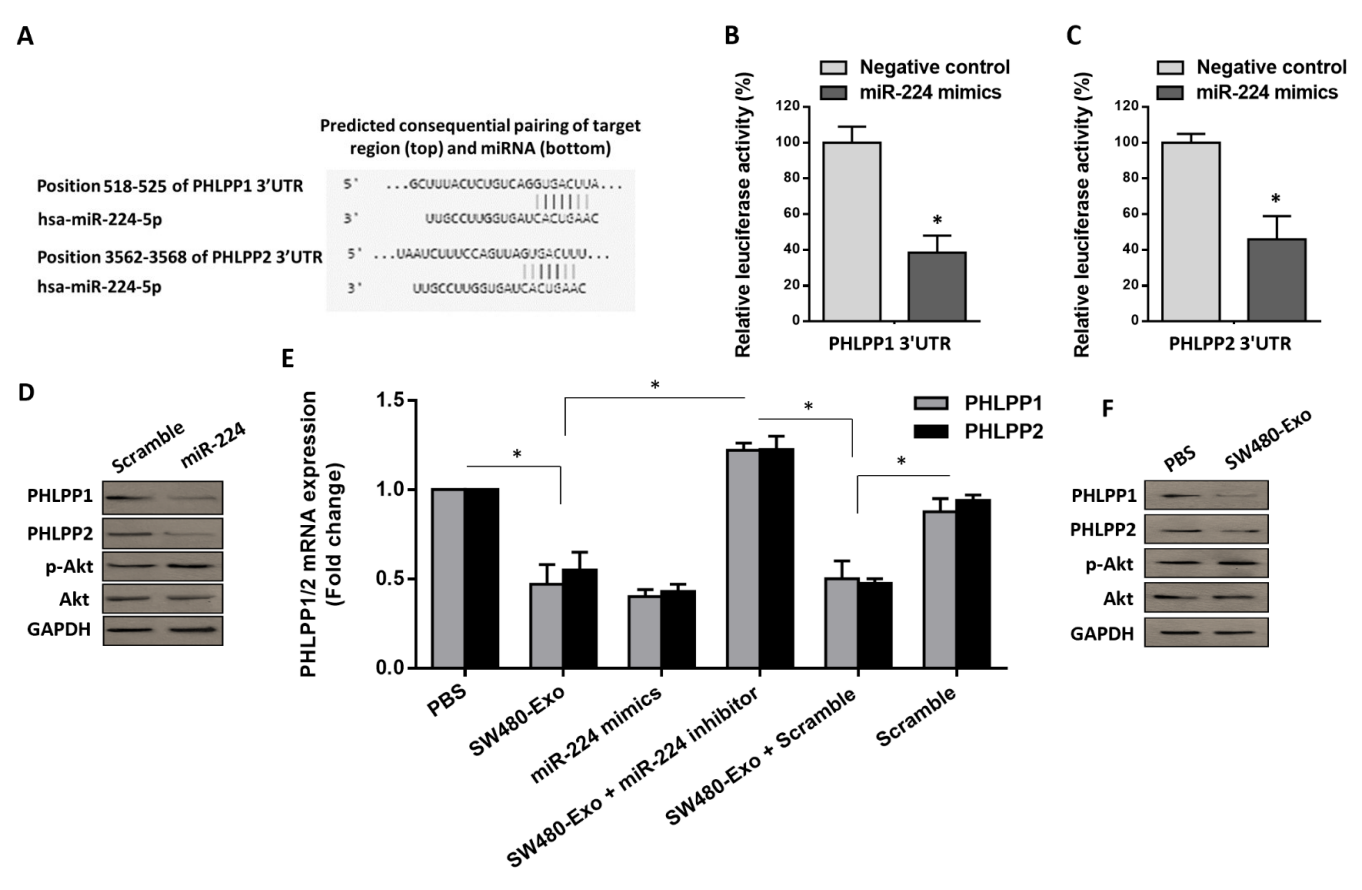
CRC-secreted exosomal miR-224 regulates Akt signaling through targeting PHLPP isoforms in activated fibroblasts. A. The specific binding sites between miR-224 and 3′-UTR of PHLPP1 and PHLPP2 are predicted by TargetScan 7.2 (http://www.targetscan.org). B, C. Luciferase activities in fibroblasts, 24 h after co-transfection with a 3′-UTR of PHLPP1 or PHLPP2 luciferase reporter construct and miR-224 mimics or negative control. miR-224 suppresses luciferase activity in the cells that were transfected by PHLPP 3′-UTR luciferase constructs (*P *< 0.05), suggesting that PHLPP1 and PHLPP2 are directly targeted by miR-224. D. Western blot analysis indicated that enforced expression of miR-224 caused a significant reduction in the protein levels of PHLPP1 and PHLPP2, and triggered Akt phosphorylation in fibroblasts. E. The mean normalized ratios for PHLPP1 and PHLPP2 transcript levels were measured by RT-qPCR. Results revealed that the transcript levels were significantly decreased in fibroblasts, 48 h following incubation with 100 μg/mL SW480 exosomes, compared to negative control. Although the enforced expression of miR-224 in fibroblasts recapitulated the inhibitory effects of SW480-Exo on the transcript levels of PHLPP1 and PHLPP2, inhibiting miR-224 abrogated the inhibiting effects of SW480 exosomes on the PHLPP1 and PHLPP2 transcript levels in fibroblasts. F. Western blot analysis revealed that incubation of fibroblasts with 100 µg/mL SW480 exosomes led to a considerable decrease in the protein levels of PHLPP1 and PHLPP2, and enhanced the phosphorylation of Akt, 48 h after incubation. Actin was used as an endogenous loading control. Western blot images are representative of at least three independent experiments. Columns, mean of three different experiments; bars, SD. *P-value < 0.05.

Considering a relatively higher amount of miR-224 in exosomes derived from metastatic CRC cells (Figure 4A(Fig. 4)) and its clinical significance (Figure 2(Fig. 2)), we sought to investigate the underlying mechanism by which exosomal miR-224 can do during CRC pathogenesis. As the first step, we employed bioinformatics exploration to identify potential target genes of this miRNA. Among different targets, we focused on the phosphatase family members PHLPP1 and PHLPP2, as they have been identified as negative regulators of Akt signaling (Brognard et al., 2007[8]; Lee and Kim, 2020[33]). Since it was revealed that PHLPP1 and PHLPP2 both dephosphorylate the same residue (hydrophobic phosphorylation motif) on Akt and consequently terminate Akt signaling, miRTargetLink Human algorithm was utilized to show an integrated network between these genes and any possible targeting miRNAs. By considering strong experimental evidence, several possible miRNAs including miR-224 were identified (Supplementary information, Figure 3). Moreover, the specific binding sites between miR-224 and the 3'-UTR of both PHLPP1 and PHLPP2 were predicted by TargetScan 7.2 (Figure 5A(Fig. 5)). To verify whether PHLPP isoforms can be directly targeted by miR-224-3p in fibroblasts, we constructed a luciferase reporter plasmid containing the Renilla luciferase encoding gene fused with either the 3'-UTR of PHLPP 1 or PHLPP 2. The construct was co-transfected with either miR-224-3p mimic or a negative control into fibroblasts. The luciferase activity of the PHLPP 1/2 3'-UTR construct was then measured using a dual-luciferase reporter assay. Our findings demonstrated that miR-224-3p specifically suppressed the luciferase activity of the PHLPP 1/2 3'-UTR reporter construct in fibroblasts (Figure 5B, C(Fig. 5)). These findings showed that PHLPP 1/2 are direct targets of miR-224. To answer this question that whether miR-224 affects the PHLPP 1/2 expression levels, we transfected fibroblasts with either miR-224 mimic or scramble; thence, the protein levels of PHLPP 1/2 were analyzed. Importantly, results showed that when miR-224 was overexpressed, a significant reduction in the protein levels of PHLPP 1/2 was detected (Figure 5D(Fig. 5)), indicating that miR-224 may regulate PHLPP 1/2 in fibroblasts. Additionally, to better grasp the functional importance of PHLPP 1/2 in inhibiting Akt signaling, we measured the levels of phosphorylated forms of Akt (pAkt) in fibroblasts that were transfected with miR-224 mimic than the cells that were transfected with scramble. Western blot analysis revealed a significant activation of Akt in fibroblasts transfected with miR-224 mimic, as the phosphorylation of this protein was induced 48 h after transfection (Figure 5D(Fig. 5)). 

Activation of Akt signaling is involved in the stromal reprogramming to CAFs in TME (Fang et al., 2023[17]). Given the evidence of miR-224-5p directly targeting PHLPP 1/2, we speculated on whether CRC-secreted miR-224 could affect Akt signaling in stromal fibroblasts. As a gradual increase in the miR-224 level was observed in SW480-Exo-treated fibroblasts (Figure 3B(Fig. 3)), we expanded our study to measure the expression levels of PHLPP 1/2 following treatment of fibroblasts with 100 µg/mL SW480-Exo. RT-qPCR results indicated a significant decrease of in the transcript levels of PHLPP 1/2 in SW480 exosome-activated fibroblasts (Figure 5E(Fig. 5)). In order to attribute a functional role for SW480 CRC-secreted miR-224, stromal cells were transfected with miR-224 inhibitors. As expected, pre-transfection of fibroblasts with miR-224 inhibitor antagonized the inhibitory effects of SW480 CRC-Exo on the PHLPP1/2 transcript levels after 48 h (Figure 5E(Fig. 5)). In line with this, western blot analysis indicated that SW480 CRC-Exo (100 µg/mL) caused a considerable decrease in the protein levels of PHLPP1/2, while the treatment triggered Akt phosphorylation in fibroblasts (Figure 5F(Fig. 5)). Altogether, these data attribute an inhibitory role to PHLPP 1/2 whereby it inhibits Akt signaling; we also underscore a supporting model in which CRC-secreted exosomal miR-224 adjusts Akt signaling by regulating PHLPP 1/2.

### Activated stromal fibroblasts may promote endothelial cell angiogenesis; a possible proangiogenic role for CRC-secreted miR-224

**Figure 6:**
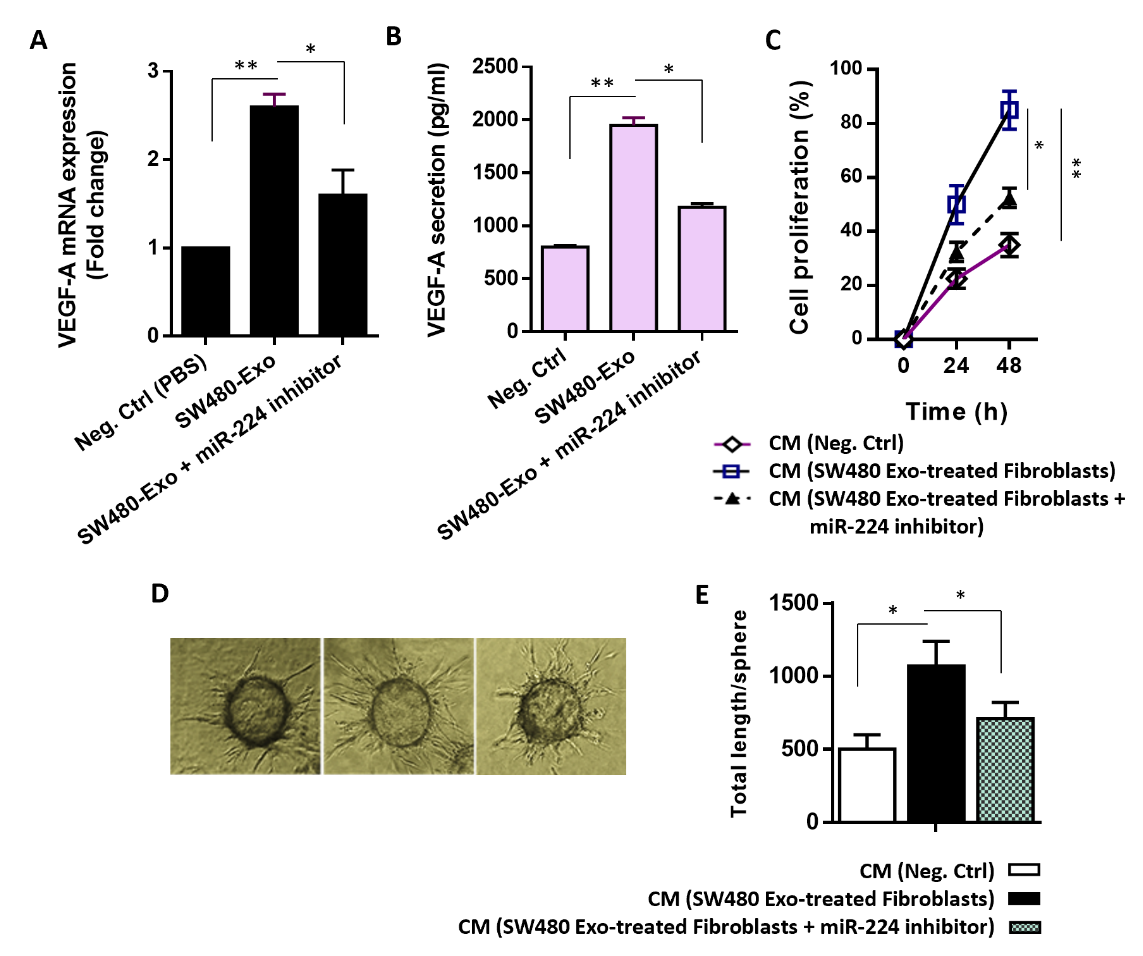
Exosomal transfer of miR-224 from CRC cells plays a role in endothelial cell angiogenesis. A. The mean normalized ratios for VEGF mRNA levels, measured by RT-qPCR indicated that treatment by SW48-Exo (100 µg/mL) caused a significant up-regulation of VEGF transcript levels in fibroblasts after 48 h. However, when fibroblasts were transfected with miR-224 inhibitor, the promoting effect of exosomes on the VEGF transcript level was partially reduced. B. Protein secretion level of VEGF, assessed by ELISA indicated that although SW480 CRC-Exo (100 μg/mL) exhibited an enhancing effect on the secretion of VEGF protein from activated stromal fibroblasts, this effect was partially diminished when fibroblasts were transfected with miR-224 inhibitor. C. Conditioned media (CM) of CRC exosome-activated fibroblasts had promoting effect on the proliferation rate of HUVECs at time points 24 and 48 h. As expected, when fibroblasts were transfected with miR-224 inhibitor, the inductive effect of CAF on endothelial cell angiogenesis was remarkably reduced at the indicated time points. D, E. Promoting effects of the conditioned media prepared from fibroblasts under different conditions on endothelial cell sprouting. D. Representative micrographs of angiogenic outgrowth of different treatments (Left panel: CM derived from stromal control cells; middle panel: CM derived from fibroblasts incubated with 100 mg/mL SW480-Exosomes; and Right panel: CM derived from SW480-Exo-treated fibroblasts transfected with miR-224 inhibitor). Results showed that CM derived from fibroblasts incubated with 100 mg/mL SW480 exosomes quantitatively enhanced HUVEC sprouting while transfecting endothelial cells with miR-224 inhibitor impeded these proangiogenic effects to some extent. E. Quantitative analysis of sprout length of spheroids proposed that the angiogenic effect of activated fibroblasts may mediate by the exosomal shuttling of miR-224 from aggressive CRC cells. Columns or points, mean of three different experiments; bars, SD. *P-value < 0.05, **P-value < 0.01.

One of the different mechanisms through which CAFs promote tumor growth and progression is inducing angiogenesis through recruitment of endothelial cells (Joshi et al., 2021[27]). In order to determine whether CAFs expressed factors capable of promoting angiogenesis, we measured the transcript levels of VEGF in fibroblasts incubated by 100 μg/mL SW480-Exo. As shown in Figure 6A(Fig. 6), RT-qPCR results revealed a significant increase in the mRNA levels of VEGF in SW480-Exo-activated fibroblasts, 48 h after incubation, than in stromal control cells. Consistent with this, we found that the SW480 CRC-Exo (100 μg/mL) exhibited an enhancing effect on the secretion of VEGF protein from activated stromal fibroblasts (Figure 6B(Fig. 6)). Importantly, the promoting effect of SW480 CRC-Exo on the transcript and secretion levels of VEGF was partially diminished when fibroblasts were transfected with miR-224 inhibitor (Figure 6A, B(Fig. 6)). 

Additionally, to show the functional significance of changes in VEGF secretion caused by SW480 CRC exosomes on endothelial cell angiogenesis, the proliferation rate and vascular behavior of endothelial cells *in vitro* were evaluated. Our observations showed that when HUVECs were incubated with the conditioned media derived from SW480-Exo-activated fibroblasts, the proliferation of cells was enhanced (Figure 6C(Fig. 6)), and the ability of endothelial cells to sprout was significantly improved (Figure 6D, E(Fig. 6)). However, when fibroblasts were transfected with miR-224 inhibitor, the inductive effect of CAF on endothelial cell angiogenesis was remarkably reduced (Figure 6C-E(Fig. 6)). These findings led us to propose that the exosomal transfer of miR-224 from aggressive CRC cells may take part in the stromal reprogramming to CAFs in a way that these activated fibroblasts can promote endothelial cell angiogenesis. 

## Discussion

Circulating miRNAs have been identified as promising diagnostic biomarkers that provide unique insights and a more dynamic perspective on tumor progression (Maminezhad et al., 2020[41]; Wang et al., 2018[58]). In this study, we utilized prior research findings and leveraged available cancer databases, such as dbDEMC 3.0 (Xu et al., 2022[63]), which encompass miRNA expression patterns in human tumors from Gene Expression Omnibus and The Cancer Genome Atlas databases. By employing this approach, we nominated six miRNAs that are likely involved in signaling pathways related to the pathogenesis of CRC. Accordingly, we established a panel of six-circulating miRNA signature including miR-103a-3p, miR-135b-5p, miR-182-5p, miR-215-5p, miR-224-5p, and miR-455-5p and found that their expression levels could accurately discriminate CRC patients from healthy controls. Given the fact that the majority of circulating miRNAs originate from tumor tissue (Cancer Genome Atlas Network Network, 2012[10]), determining their levels in exosomes as transferring structures for miRNAs, can serve as a way to trace them back to tumors and therefore reflect certain properties of the tumor (Möller and Lobb, 2020[46]). As circulating miRNAs seem to be mainly associated to exosomes (Valadi et al., 2007[57]), we first quantified the levels of the candidate miRNAs in exosomes derived from two different CRC cell lines (SW480 and Caco2) which exhibit distinct pattern of metastatic spread. In light of the clinical significance of miR-224 as a possible diagnostic biomarker and the notable increase in its levels found in exosomes derived from metastatic SW480 CRC cells, this miRNA was selected for further functional studies.

The stromal microenvironment is mainly composed of a heterogeneous population of activated fibroblasts, which undergo many transcriptional and functional changes during tumor development and progression (Kalluri, 2016[28]). Despite the mutual communications between tumor cells and resident fibroblasts, there are no significant genetic differences between CAFs and normal fibroblasts. This suggests that oncogenic mutations likely do not play a major role in the acquisition of the CAF phenotype (Corver et al., 2011[14]). Compared to their quiescent counterparts, activated CAFs exhibit malignant biological behaviors as they grow more rapidly and possess greater capabilities for migration, metabolic activity, and secretion of cytokines, chemokines and growth factors. This differences may be due to stromal reprogramming that occurs in CAFs as a result of their activation within the TME (Mao et al., 2021[43]). The underlying mechanisms that activate normal quiescent fibroblasts to become CAFs are complicated and require further investigation. Generally, the dynamic crosstalk between tumor and resident stromal cells can activate the fibroblasts. This activation may be mediated by growth factors, such as TGF-β, released by tumor cells (Kojima et al., 2010[29]). Recent studies have proven that the interplay between tumor and stromal cells within the TME is mainly mediated by secreting exosomes. The pleiotropic capacity of tumor-derived exosomes in activating resident fibroblasts contributes to promoting tumor growth, neovascularization, immune escape, and metastasis (Giusti et al., 2022[21]; Yeon et al., 2018[67]). Recently, it has been shown that exosomes released by ovarian tumor cells can educate normal fibroblasts into an active state; the event, in turn, is able to remodel the tumor stroma to be more advantageous for tumor progression (Giusti et al., 2022[21]). Consistently, we demonstrated that exosomes released by metastatic CRC cells take part in the stromal reprogramming to CAFs. Activated CAFs exhibited a higher proliferation rate and were characterized by a considerable induction in the expression levels of CAF-specific markers and CAF-derived growth factor and cytokines. 

Akt signaling plays a critical role in maintaining a delicate equilibrium between cell survival and apoptosis, as well as regulating differentiation and metabolism (Martelli et al., 2010[44]). To tilt the balance towards survival, signaling molecules often bind to receptors that activate the lipid kinase phosphatidylinositol 3-kinase (PI3K). This activation leads to the production of 3'-phosphoinositide lipid second messengers, with phosphatidylinositol-3,4,5-trisphosphate being the most notable among them (Luo et al., 2003[39]). It was well-established that the phosphatase family members PHLPP1 and PHLPP2 act as negative regulators of Akt signaling (Brognard et al., 2007[8]). It has been shown that when the termination of signaling through the PI3K/ Akt pathway failed, it led to the inhibition of apoptosis and enhances tumor growth (Gao et al., 2005[18]). Recently, compelling evidence has surfaced that PHLPP1 and PHLPP2 function as tumor suppressor genes, with frequent depletion of their expression observed in various types of human cancers including CRC (Li et al., 2014[34]; O'Neill et al., 2013[48]). We identified a negative correlation between miR-224 and the protein levels of the phosphatase family members PHLPP1 and PHLPP2. Also, we confirmed that targeting of the PHLPP isoforms links miR-224 to Akt signaling in activated fibroblasts. These findings are consistent with previous studies demonstrating that PHLPPs terminate Akt signaling by directly dephosphorylating and inactivating AKT (Gao et al., 2005[18]). 

Mounting evidence revealed that the abundant miRNAs in tumor cell-derived exosomes play a crucial role in the stromal reprogramming to CAFs (Yang et al., 2019[66]). For instance, it was demonstrated that breast tumor-secreted exosomal miR-9 is able to promote the transition of normal fibroblasts toward a CAF-like phenotype. Upon exosome-mediated delivery of miR-9, a signature of different genes correlated with cell motility and extracellular matrix organization was identified (Baroni et al., 2016[5]). The contribution of hepatocellular cancer-derived exosomal miR-21 in converting hepatic stellate (stromal) cells to CAFs has been explored in hepatocellular carcinoma. It was revealed that exosomal miR-21 secreted from liver cancer cells mediates hepatic stromal cell activation through modulating the PTEN/Akt signaling axis. Besides, activated hepatic stromal cells exhibited an aberrant activation of lipogenesis and induced the expressions of various factors related to angiogenesis and tumor progression in HCC. In melanoma, exosomal miR-155 was found to trigger a proangiogenic switch in CAFs by targeting the suppressor of cytokine signaling 1 and mediating STAT3 activation (Zhou et al., 2018[70]). 

Our study revealed that exosomes carrying miR-224 released by metastatic CRC cells interact with the stromal-derived fibroblasts and contribute to their activation, as demonstrated by increased stromal cell proliferation and migration as well as inducing CAF-specific markers and CAF-derived cytokines. After validating PHLPP 1/2 as authentic targets of miR-224, we expanded our study to uncover the precise mechanisms through which CRC-secreted miR-224 affect the malignant behavior of activated stromal fibroblasts. We indicated that exosomal miR-224 released by metastatic SW480 CRC cells, but not non-metastatic Caco2 cell, regulates PHLPP phosphatases, thus activates Akt signaling. Importantly, overexpression of miR-224 recapitulated the promoting effects of SW480 exosomes on Akt signaling; inhibiting miR-224 was sufficient to rescue PHLPPs from exosome-induced down-regulation in stromal cells. 

CAF activation can bring about substantial alterations in the TME, resulting in transcriptional modifications driven by various signaling pathways (Wu et al., 2021[62]). Akt signaling can impact tumor aggressiveness by affecting the EMT (Xu et al., 2015[64]). As targeting PHLPP phosphatases links miR-224 to the oncogenic Akt signaling in activated stromal fibroblasts, we investigated the effects of CRC-secreted exosomal miR-224 in inducing malignant biological behaviors of CAFs. It was demonstrated that various transcription factors such as HIF-1α, Snail, and Twist, constitute complex regulatory networks to modulate the formation, activation, and malignant phenotype of CAFs in the TME (Fang et al., 2023[17]). Consistently, we observed that CRC-secreted exosomal miR-224 caused up-regulation of the EMT-transcription factors, thereby promoting the proliferation and migration capabilities of activated fibroblasts. Although angiogenesis is influenced by various factors, VEGF is recognized as a predominant inducer of physiological and pathophysiological angiogenesis (Hoeben et al., 2004[24]). The expression of VEGF is regulated by the transcription factor HIF-1α through binding to a hypoxia response element within its promoter (Manalo et al., 2005[42]). VEGF and HIF-1α are closely linked to poor survival rates in CRC, making them crucial factors to consider in understanding tumor progression (Cao et al., 2009[11]). Emerging evidence has proven that CAFs actively contributes to vascular growth through secreting soluble signaling factors such as VEGF (Sewell-Loftin et al., 2017[51]). In line with this, we observed that once activated by CRC exosomes, CAFs are able to induce endothelial cell proliferation and sprouting through their secretome containing a higher concentration of VEGF. 

## Conclusion

Circulating miRNAs have been identified as promising liquid biopsy biomarkers, having the potential to improve the diagnostic profile of human cancers (Wang et al., 2018[58]). The present study unveiled the potential of circulating miRNAs as non-invasive biomarkers for CRC diagnosis. As we delve deeper into the biology of TME, it is becoming evident that quiescent fibroblasts probably give rise to a heterogeneous population of activated fibroblasts. Transition of stromal cells into an active state alters the transcriptional and secretory characteristics, resulting in a remodulation of the TME and impact the malignant phenotype. Our findings provided the first evidence that exosomal transfer of miR-224 from metastatic CRC cells trigger stromal reprogramming to CAFs through targeting PHPPL phosphatases and mediating Akt activation. Also, we found that CRC-secreted exosomal miR-224 induced proangiogenic switch in activated fibroblasts that elicit a vascular response in endothelial cells (Figure 6(Fig. 6)). These findings offer valuable insights into the regulatory networks implicated in the development of CRC and suggest that approach intervening the stromal reprogramming to CAFs may have a potential application in targeted therapies against CRC.

## Declaration

### Acknowledgments

The authors appreciate the contribution of all individuals who participated in this study. We also thank Dr. Katayoon Pakravan for her excellent help and advice during the entire study. 

### Conflict of interest

The authors declare that they have no conflict of interest.

### Funding

This work was supported partially by the Islamic Azad University of Tehran Medical Sciences, Iran.

### Author Contributions

S.B. conceived and designed the study. F.K., F.A.B., R.R., and A.F. performed the experiments. F.A.B., A.F. and F.R. contributed to the provision of study materials or clinical samples. F.K. and E.R. drafted the manuscript, and S.B. and E.R. contributed to revising it. S.B. supervised the study and provided administrative support. All authors reviewed the final manuscript.

### Data availability 

The data supporting the results of this study are attached to the text as Supplementary data.

### Ethical approval 

The study was conducted according to the guidelines of the Declaration of Helsinki and approved by the ethics committee of Islamic Azad University, Tehran, Iran.

## Supplementary Material

Supplementary information

Supplementary data
