# Supplementary material for: Exosomes derived from colorectal cancer cells take part in activation of stromal fibroblasts through regulating PHLPP isoforms
Source: EXCLI J. 2024 May 2;23:634–54. doi: 10.17179/excli2024-6926 (PMC11180944; doi:10.17179/excli2024-6926)
Supplement: Supplementary information [file EXCLI-23-634-s-001.pdf]

EXCLI Journal 2024;23:634-654 – ISSN 1611-2156
Received: December 30, 2023, accepted: April 12, 2024, published: May 02, 2024

Supplementary information to:

Original article:

EXOSOMES DERIVED FROM COLORECTAL CANCER CELLS TAKE PART IN ACTIVATION OF STROMAL FIBROBLASTS THROUGH REGULATING PHLPP ISOFORMS

Fatemeh Khaloozadeh[1], Ehsan Razmara[2], Fatemeh Asgharpour-Babayian[3], Alireza Fallah[3], Reihaneh Ramezani[4], Fatemeh Rouhollah[1,*], Sadegh Babashah[3,5]

1 Department of Cellular and Molecular Biology, Faculty of Advanced Sciences and Technology, Tehran Medical Sciences, Islamic Azad University, Tehran, Iran
2 Department of Medical Genetics, Faculty of Medical Sciences, Tarbiat Modares University, Tehran, Iran
3 Research and Development Center of Biotechnology, Tarbiat Modares University, Tehran, Iran
4 Department of Family Therapy, Women Research Center, Alzahra University, Tehran, Iran
5 Department of Molecular Genetics, Faculty of Biological Sciences, Tarbiat Modares University, Tehran, Iran

* **Corresponding author:** Fatemeh Rouhollah, Ph.D., Department of Cellular and Molecular Biology, Faculty of Advanced Sciences and Technology, Tehran Medical Sciences, Islamic Azad University, Tehran, Iran.
E-mail: f.rouhollah@yahoo.com; frohollah@iautmu.ac.ir

https://dx.doi.org/10.17179/excli2024-6926

This is an Open Access article distributed under the terms of the Creative Commons Attribution License (http://creativecommons.org/licenses/by/4.0/).

EXCLI Journal 2024;23:634-654 – ISSN 1611-2156
Received: December 30, 2023, accepted: April 12, 2024, published: May 02, 2024

Supplementary Table 1: The clinicopathological characteristics of CRC patients

Characteristics	No. of CRC patients (%)	No. of HC individuals (%)
**Age (years)**		
<50	15 (37.5)	12 (40)
≥50	25 (62.5)	18 (60)
**Gender**		
Male	32(80)	22(73)
Female	8 (20)	8 (27)
**Position**		
Colon	17 (42.5)	
Rectal	23 (57.5)	
**Tumor size**		
<2 cm	14 (35)	
≥2 cm	26 (65)	
**LNM**		
Metastasis	17 (42.5)	
No metastasis	23 (57.5)	
**Tumor stage**		
I+II	24 (60)	
III	16 (40)	

Abbreviations: CRC, Colorectal cancer; HC, Healthy controls; LNM, Lymph node metastasis

EXCLI Journal 2024;23:634-654 – ISSN 1611-2156
Received: December 30, 2023, accepted: April 12, 2024, published: May 02, 2024

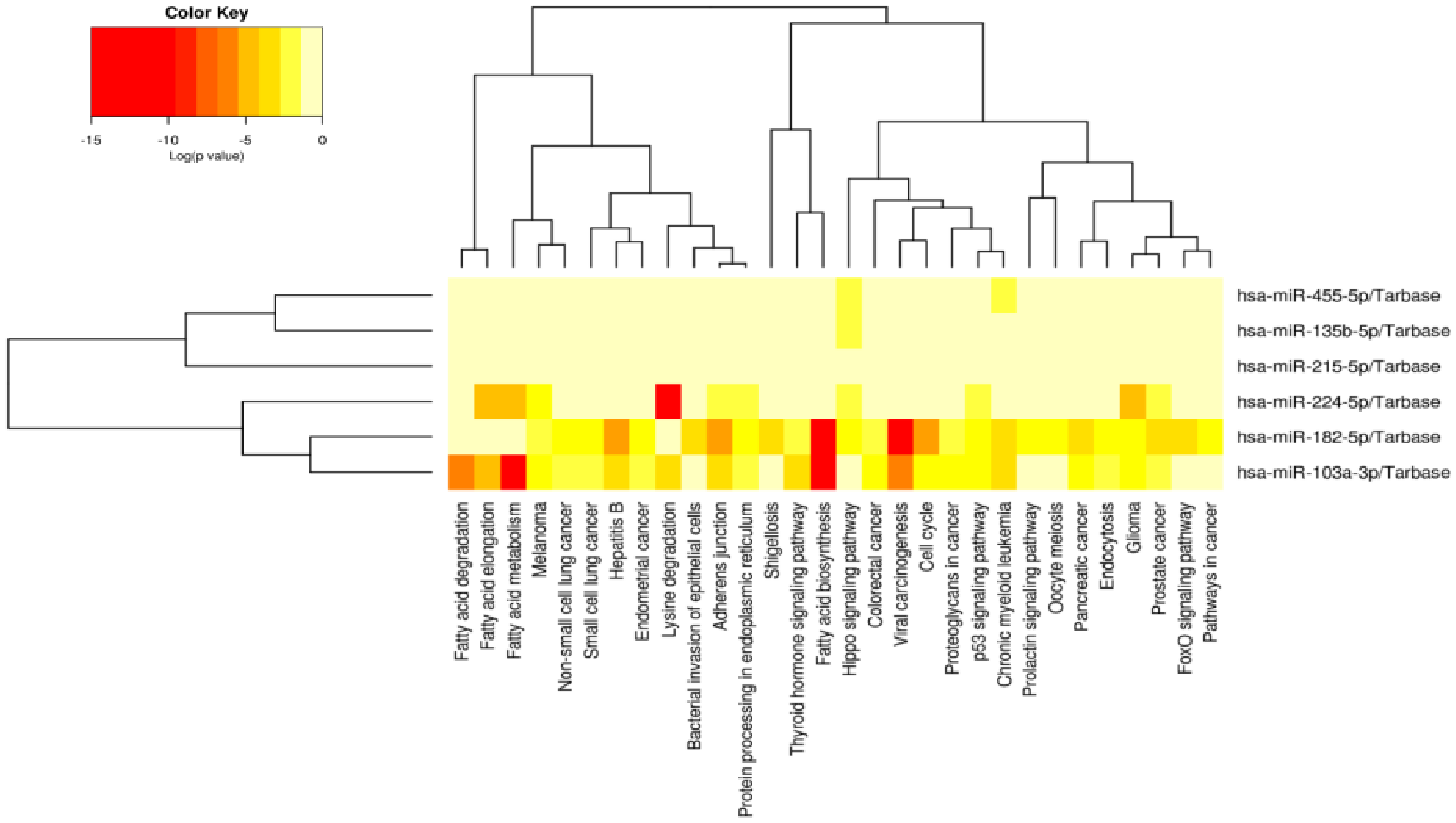

Supplementary Figure 1: Functional enrichment analysis. The heatmap illustrates the comparison of differentially expressed candidate miRNAs with significantly enriched functional pathways. In the heatmap, reddish colors indicate higher statistical significance, as denoted by the color key provided above. Additionally, the attached dendrograms on both axes represent the results of hierarchical clustering for miRNAs and pathways, respectively. The figure was generated using the output from Diana miRpath V.3.0 (https://dianalab.e-ce.uth.gr/html/mirpathv3/index.php?r=mirpath)

EXCLI Journal 2024;23:634-654 – ISSN 1611-2156
Received: December 30, 2023, accepted: April 12, 2024, published: May 02, 2024

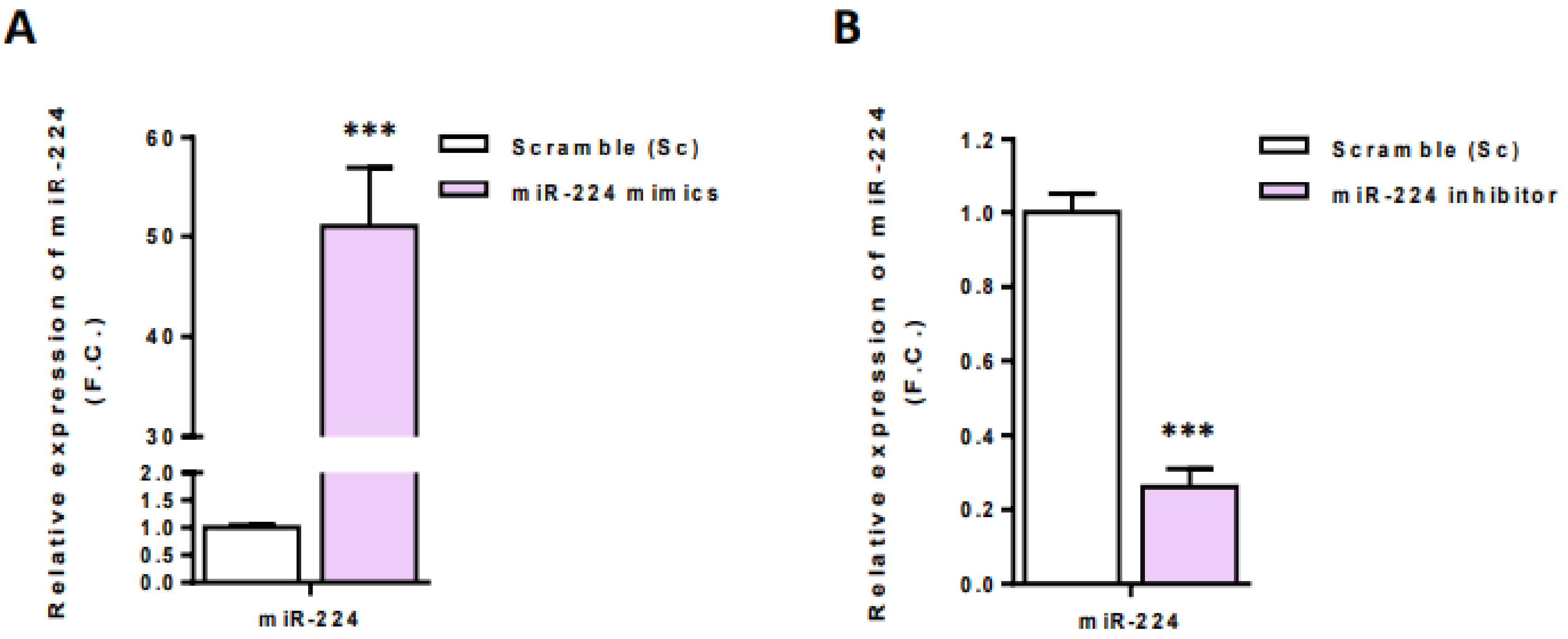

Supplementary Figure 2: Transfection efficiency of miR-224 mimic and miR-224 inhibitor measured by RT-qPCR in stromal cells, following 24h after transfection. **A.** RT-qPCR data revealed that miR-224 expression was substantially increased in fibroblasts that were transfected by miR-224 mimic than the cells transfected with negative control scramble. **B.** Results also demonstrated a significant decrease in miR-224 expression level in fibroblasts transfected with miR-224 inhibitor than those cells transfected with negative control scramble. Columns, mean of three different experiments; bars, SD. ** *P*< 0.01

EXCLI Journal 2024;23:634-654 – ISSN 1611-2156
Received: December 30, 2023, accepted: April 12, 2024, published: May 02, 2024

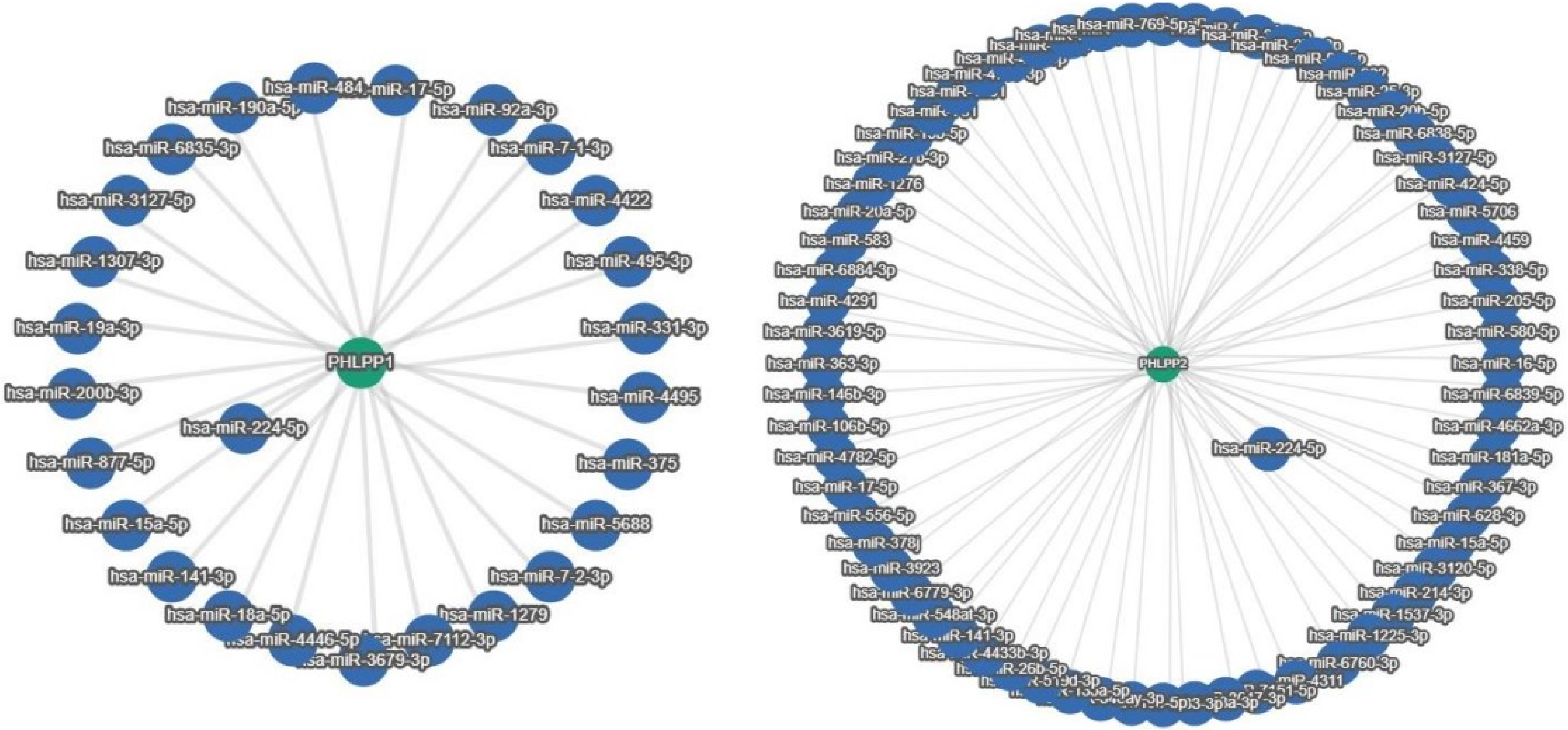

Supplementary Figure 3: The interaction networks between PHLPP1 and PHLPP2 and the putative targeting miRNAs provided by miRTargetLink Human algorithm (https://ccb-web.cs.uni-saarland.de/mirtargetlink)
